# Insights and Challenges in Correcting Force Field Based Solvation Free Energies Using a Neural Network Potential

**DOI:** 10.1021/acs.jpcb.4c01417

**Published:** 2024-07-08

**Authors:** Johannes Karwounopoulos, Zhiyi Wu, Sara Tkaczyk, Shuzhe Wang, Adam Baskerville, Kavindri Ranasinghe, Thierry Langer, Geoffrey P. F. Wood, Marcus Wieder, Stefan Boresch

**Affiliations:** † Faculty of Chemistry, Institute of Computational Biological Chemistry, University Vienna, Währingerstr. 17, 1090 Vienna, Austria; ‡ Vienna Doctoral School of Chemistry (DoSChem), University of Vienna, Währingerstr. 42, 1090 Vienna, Austria; ¶ Exscientia plc, Schroedinger Building, Oxford OX4 4GE, United Kingdom; § Department of Pharmaceutical Sciences, Pharmaceutical Chemistry Division, University of Vienna, Josef-Holaubek-Platz 2, 1090 Vienna, Austria; ∥ Vienna Doctoral School of Pharmaceutical, Nutritional and Sport Sciences (PhaNuSpo),University of Vienna, Josef-Holaubek-Platz 2, 1090 Vienna, Austria; ⊥ Open Molecular Software Foundation, Davis, California 95616, United States

## Abstract

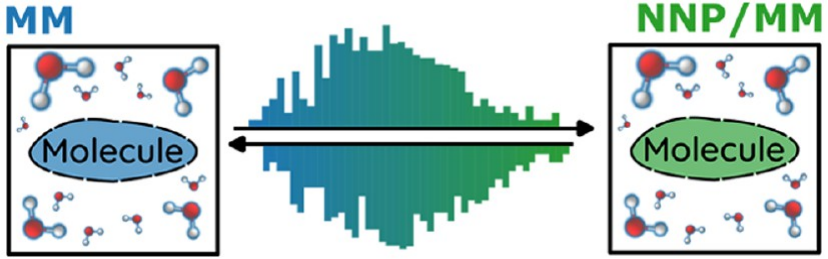

We present a comprehensive study investigating the potential gain in accuracy for calculating absolute solvation free energies (ASFE) using a neural network potential to describe the intramolecular energy of the solute. We calculated the ASFE for most compounds from the FreeSolv database using the Open Force Field (OpenFF) and compared them to earlier results obtained with the CHARMM General Force Field (CGenFF). By applying a nonequilibrium (NEQ) switching approach between the molecular mechanics (MM) description (either OpenFF or CGenFF) and the neural net potential (NNP)/MM level of theory (using ANI-2x as the NNP potential), we attempted to improve the accuracy of the calculated ASFEs. The predictive performance of the results did not change when this approach was applied to all 589 small molecules in the FreeSolv database that ANI-2x can describe. When selecting a subset of 156 molecules, focusing on compounds where the force fields performed poorly, we saw a slight improvement in the root-mean-square error (RMSE) and mean absolute error (MAE). The majority of our calculations utilized unidirectional NEQ protocols based on Jarzynski’s equation. Additionally, we conducted bidirectional NEQ switching for a subset of 156 solutes. Notably, only a small fraction (10 out of 156) exhibited statistically significant discrepancies between unidirectional and bidirectional NEQ switching free energy estimates.

## Introduction

The importance of solvation in biological processes cannot be overestimated. Among other things, the correct description of the interaction of water with biological macromolecules and small molecule substrates is crucial for understanding ligand binding and, therefore, for computational techniques to predict binding affinities.^1−4^ The accuracy of the molecular-mechanical force field used in free energy difference calculations is one of the limitations of such methods. For this reason, several large-scale studies have focused on the computation of solvation free energy differences.^5−8^ The comparison of the predictions with experimental data helps to identify the strengths and weaknesses of the force field used.

Recently, we calculated solvation free energy differences for most of the compounds in the FreeSolv database using the CHARMM General force field (CGenFF).^9^ We have now completed analogous calculations with the Open force field (OpenFF), and these results are presented in detail below. Overall, the agreement with the experimental data is acceptable (for OpenFF 2.0 root mean squared error (RMSE) = 1.33 kcal/mol, mean absolute error (MAE) = 1.01 kcal/mol; for CGenFF RMSE = 1.76 kcal/mol, MAE: 1.12 kcal/mol). However, especially for CGenFF, there are a sizable number of molecules with significant discrepancies between the computed and experimentally determined solvation free energies.

Neural network potentials (NNP) are a recent development that allow a more accurate description of intra- and intermolecular interactions at an affordable cost. The use of NNPs in free energy simulations may, therefore, improve the accuracy of such calculations.^10−13^ However, although NNPs are fast compared to quantum chemical calculations, they are significantly slower than classical mechanical force fields.^14^ Furthermore, it is unknown how to apply certain “tricks” used in free energy simulations, such as soft-core potentials,^15−17^ in combination with NNPs. One can avoid both complications by indirect pathways, frequently used to compute free energy differences with quantum mechanics (QM)/molecular mechanics (MM) hybrid potential energy functions.^18−26^ Indirect free energy calculations use a computationally cheaper description of the potential energy (e.g., an MM force field) and calculate the free energy contribution needed for changing to a more expensive description of the potential energy (e.g., a QM/MM potential). The calculation of the free energy differences between the different levels of theory was shown to be nontrivial.^23,27−31^ One way to calculate them reliably is through nonequilibrium switching techniques (NEQ).^32−36^ NNPs offer a tempting trade-off between accuracy and speed compared to MM and QM methods, which is why they can be applied as the high-level potential in such indirect cycles. An early example of using NNPs to refine classical free energy simulations is a study by Rufa et al.^10^ Recently, we investigated the convergence of the correction step required in indirect pathways, i.e., calculating the free energy difference between an MM and an NNP representation of a system.^37^ In both studies, the ANI-2x^38,39^ NNP was used. Simulations with ANI and hybrid ANI/MM simulations can be carried out efficiently using TorchANI(40) and NNPOPS.^14^

The computational framework of ref (37) is suitable for use in the gas phase and aqueous solution. In this work, we explore whether MM → NNP and MM → NNP/MM corrections can improve the agreement of computed solvation free energies with the experiment. There are very efficient implementations of ANI in OpenMM; furthermore, mixing MM and ANI is straightforward.^40,41^ Therefore, the MM → NNP/MM corrections can be integrated smoothly into our automated workflows to compute the solvation free energies with CGenFF^9^ and OpenFF 2.0 (see below and the SI). Nevertheless, ANI/MM simulations are costly; therefore, we focus on the molecules that performed poorly with CGenFF, OpenFF 2.0, or both. From the ASFE results obtained at the MM level of theory, we identified the 100 compounds for each force field exhibiting the highest discrepancies compared to their experimental values (selection was limited to molecules with elemental composition covered by the ANI-2x training set; i.e.: H, C, N, O, F, S, and Cl). Thus, we created a set comprising 156 compounds, where 41 were among the worst-performing compounds for both force fields. The remaining compounds exhibited poor performance for either CGenFF (56 compounds) or OpenFF (59 compounds).

Furthermore, one must consider the limitations of the ANI/MM hybrid potential function currently implemented in OpenMM-ML. First, ANI-2x has been trained against quantum chemical calculations using the DFT functional ωB97X with the 6-31G* basis set.^39^ To improve solvation free energies calculated by QM/MM approaches, a careful choice of the DFT method/basis set is required.^42^ Second, as pointed out in ref (14), the coupling between MM and ANI is analogous to “mechanical embedding” in QM/MM simulations. In other words, only the intramolecular interactions of the solute are described by the NNP, whereas the solute–solvent interactions remain classical. Therefore, one cannot expect improvements in all cases. Specifically, describing the solute by ANI may result in different preferred conformations (compared to the force field), which, in turn, may lead to a different solvation free energy. Thus, improvements, if any, can be expected only for larger and/or flexible solutes. These cases are of interest as they can help identify shortcomings of the force field.

The remainder of the article is organized as follows: First, we report ASFEs obtained with the OpenFF force field. For the 589 compounds from the FreeSolv database that ANI-2x can handle, we calculated end-state corrections from unidirectional nonequilibrium work (NEQ) simulations. Second, we also calculated endstate corrections for the data set of 156 poor-performing compounds for the earlier CGenFF results.^9^ The endstate corrections using CGenFF for the MM description were computed using not only unidirectional but also two-sided NEQ approaches, making it possible to gauge the reliability of the computationally cheaper one-sided method. Lastly, we briefly investigate the correlation between the magnitude of the end-state corrections and the conformational flexibility of the solutes.

## Theory

### Absolute Free Energy Calculations

**Figure 1:**
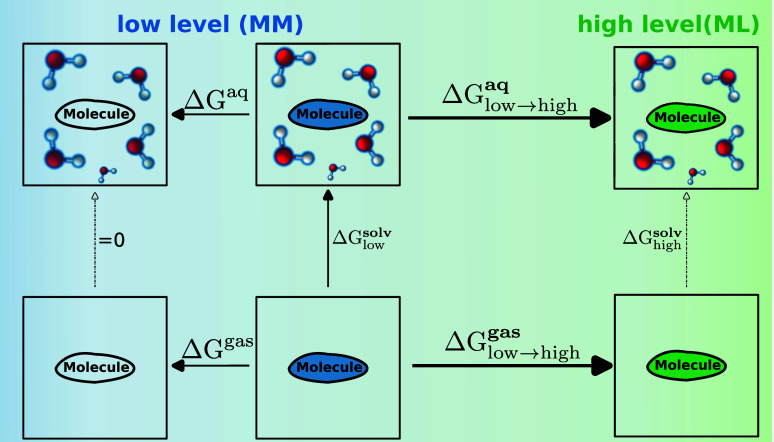
Free energy calculations between high and low levels of theory can be used to correct alchemical free energy calculations performed at a low level of theory. Left: The thermodynamic cycle used to compute an ASFE Δ*G*_low_^solv^ at the MM level of theory using annihilation of the solute’s nonbonded interactions.^43^ Right: The indirect free energy cycle to correct Δ*G*_low_^solv^ (in this work using either the CGenFF or the OpenFF force field) is from the MM to the NNP/MM level of theory.

We calculated ASFEs using the alchemical pathway shown on the left side of Figure 1. Both protocols used in this work (see the Methods section below) involve (at least partial) annihilation of the solute’s nonbonded interactions, i.e., turning off both the nonbonded intramolecular interaction of the solute and its intermolecular interactions with its surroundings (solvent).^44^ The annihilation absolute free energy protocol requires a gas phase correction; the absolute solvation free energy of interest Δ*G*_MM_^solv^ is obtained as the difference between the annihilation protocol in the gas phase Δ*G*_*L*_1__^gas^ and in solution Δ*G*_*L*_1__^aq^ (Δ*G*_MM_^solv^ = Δ*G*_*L*_1__^gas^ – Δ*G*_*L*_1__^aq^).^43^

### Endstate Correction

Free energy estimates from NEQ work values (*W*) can be calculated using the Jarzynski equation^45^ or the Crooks fluctuation theorem.^46^ The Jarzynski equation recovers the free energy estimate between a target and a reference distribution based on an NEQ work process that starts at the reference and anneals to the target distribution. According to the Jarzynski equation, the free energy difference between two states 0 and 1 (*W*_0→1_) for NEQ work distributions is obtained as follows:
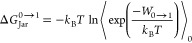
1In our specific use case, state 0 indicates the potential energy function at the low level of theory, while 1 represents the same system at a higher level of theory. The subscript 0 in <>_0_ indicates that the NEQ switching simulations to obtain the work values *W*_0→1_ are started from equilibrium configurations sampled at the lower level of theory (state 0).

The Crooks fluctuation theorem recovers the equilibrium free energy estimate between the initial and final state based on NEQ work processes that transform the reference to the target potential and *vice versa*. Thus, one has to additionally carry out sampling at and NEQ switching simulations starting from the high level of theory or, in other words, compute work values in the 1 → 0 direction. The free energy between states 0 and 1 is then given by^46^
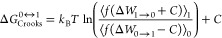
2where *f*(*x*) denotes the Fermi function 
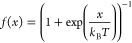
3 and
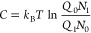
4Here, *Q* represents the canonical partition function of the respective state (0 and 1), and *N* is the number of work values in the forward and backward direction, respectively. Equation 2 is typically solved by searching iteratively for the value of *C* for which the argument of the logarithm becomes unity and, hence, the first term in eq 2 vanishes. As one sees from eq 4, the value of *C* found in this manner is essentially the sought free energy difference.

## Methods

### Overview of Calculations/Workflow

We performed ASFE calculations for most of the compounds in the FreeSolv database,^47,48^ a curated collection of experimental solvation free energies for 642 drug-like molecules. Compounds containing elements not covered in the ANI-2x training were removed, leaving 589 molecules for which an MM → NNP/MM endstate correction can be carried out. ASFEs were computed using two independent workflows, which we labeled as protocols EXS and UVIE. Here, we focus on the commonalities; additional details of each of the two protocols can be found in the SI. The UVIE protocol used the CGenFF force field^49,50^ and transformato(51,52) to calculate ASFEs at the MM level of theory. The results obtained with this approach have been previously described.^9^ In the EXS protocol, ASFEs at the MM level of theory were computed using openmmtools 0.23.0^53^ and the Open Force Field (OpenFF 2.0).^54^ While the ASFEs at the MM level of theory were calculated with different methodologies/programs, the MM → NNP/MM end-state corrections were carried out quite similarly in both approaches, though specific adaptations were necessary.

Using the EXS protocol, endstate corrections were computed for all 589 molecules, i.e., the complete subset of the FreeSolv database, excluding any compounds that contained elements not covered by the training set of ANI-2x. These corrections were calculated only unidirectionally (eq 1). MM → NNP/MM corrections using the UVIE workflow were computed for the subset of the FreeSolv database (156 molecules), for which the force field results were in poor agreement with the experimental data (cf. the Introduction). These corrections were computed by uni- and bidirectional (Crooks’ theorem, eq 2) NEQ switching methods.

### Endstate Correction with NNP

#### Equilibrium Simulations

##### MM Level of Theory

For each compound, a Langevin dynamics simulation was performed in the gas phase and in solution to generate equilibrium configurations from which the NEQ switches were started. OpenMM 8.0^41^ was used with an integration time step of 1 fs. Molecules were solvated in TIP3P^55^ water, held rigid by the SETTLE algorithm,^56^ and simulations were performed under constant pressure conditions using a Monte Carlo barostat.^57,58^ The solutes themselves were fully flexible. The treatment of nonbonded interactions was slightly different in protocols UVIE and EXS; see the SI for details. Before each simulation, the geometry of the solute was optimized by using the L-BFGS minimizer.

##### NNP/MM Level of Theory

The NNP/MM simulations were carried out completely analogously to what was just described for the MM case. The only difference is the treatment of the intramolecular energetics of the small molecule, which was calculated using the ANI-2x potential instead of the respective MM force field.^39^ Specifically, the high-performance ANI-2x potential reimplementation, NNPOPS (v.0.4), was used in this work.^14^ To interpolate between the ANI-2x and the force field, we used the OpenMM-ML package.^10^

#### Nonequilibrium Switching Simulations

NEQ switching simulations of 5 ps length were initialized by randomly selecting 300 conformations (with replacement) from the equilibrium trajectories (either at the MM or NNP/MM endstate). For the total number of conformations saved in protocols EXS and UVIE, respectively, see the Supporting Information. The NEQ protocol consisted of an alternating sequence of propagation and perturbation steps in which the potential was slowly perturbed while propagating the coordinates. In each perturbation step, the coupling parameter λ = *t*/τ was used to scale the potential energy *U* = (1 – λ) *U*_MM_ + *λ U*_NNP/MM_ as a function of the current perturbation *t* ∈ [0, τ] and the total protocol length τ. Each propagation step consisted of a 1 fs integration step to propagate conformation *x* from *x*_*t*_ to *x*_*t*+1_. The work value along a particular trajectory up to time *t* + 1 is calculated by using *W*_*t*_ = *U*_*t*+1_(*x*_*t*+1_) – *U*_*t*_(*x*_*t*+1_). Nonequilibrium switching simulations can be performed uni- and bidirectionally, i.e., employing either the Jarzynski or the Crooks equation. We used the exponential averaging (EXP) estimator and the Bennett Acceptance Ratio (BAR) estimator (both as implemented in pymbar(59)) to obtain free energies from unidirectional and bidirectional NEQ switching simulations, respectively. Errors were estimated via a bootstrapping procedure: Out of the pool of 300 work values, we randomly selected a subset (with replacement) for which Δ*G*_MM→NNP_ was computed. This procedure was repeated 1000 times, and the standard deviation obtained in this manner was used as the error estimate.

NEQ switching simulations were performed both in aqueous solution and in the gas phase to obtain Δ*G*_MM → NNP/MM_^aq^ and Δ*G*_MM → NNP_^gas^ (see the right-hand side of Figure 1). The free energy difference (Δ*G*_MM→NNP/MM_) between levels of theory and thus the endstate correction value is obtained by Δ*G*_MM → NNP/MM_^corr^ = −Δ*G*_MM → NNP_^gas^ + Δ*G*_MM → NNP/MM_^aq^. Thus, the corrected ASFE can be calculated as Δ*G*_NNP/MM_^solv^ = Δ*G*_MM_^solv^ + Δ*G*_MM → NNP/MM_^corr^.

In the EXS protocol, corrections were computed using only unidirectional NEQ switching simulations, limiting the calculation of the free energy difference to the EXP estimator. By contrast, using protocol UVIE, also bidirectional NEQ calculations were performed, allowing the use of Crooks’ equation. Corrections obtained by Crooks’ equation are denoted as Δ*G*_MM ↔ NNP/MM_^corr, Crooks^. Results are presented as the deviation of the computed result from the experimental reference value, i.e.,
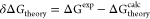
5

The superscripts *exp* and *calc* denote the experimental and calculated solvation free energy, respectively. The subscript *theory* stands for either OpenFF or CGenFF at the MM level or the ANI-2x corrected result, indicated as OpenFF/ANI or CGenFF/ANI, respectively.

#### Multistate Equilibrium Free Energy Simulations

For 10 molecules out of the 156 molecule subset, we performed multistate equilibrium free energy simulations (MFES) using 11 equidistant λ windows (λ = 0.1, 0.2, ..., 1.0), with λ = 0 being the MM-endstate and λ = 1 being the NNP-endstate (protocol UVIE only). Sampling was performed for 5 ns from each equilibrium distribution, and 5,000 samples were collected. To ensure that the samples represent the stationary distribution, the initial 20% of each simulation was discarded, resulting in 4,000 samples per simulation and λ window. These were further pruned, and only every fifth sample was used to calculate the free energy difference of interest. From the combined set of 11 alchemical states, consisting of 800 samples each (11 × 800 in total), connecting the MM and NNP potentials, we calculated the free energy difference using the MBAR estimator, as implemented in the pymbar package.^59^ We monitored whether there was overlap between neighboring λ-states.

## Results and Discussion

### ASFE Results Using OpenFF 2.0

**Figure 2:**
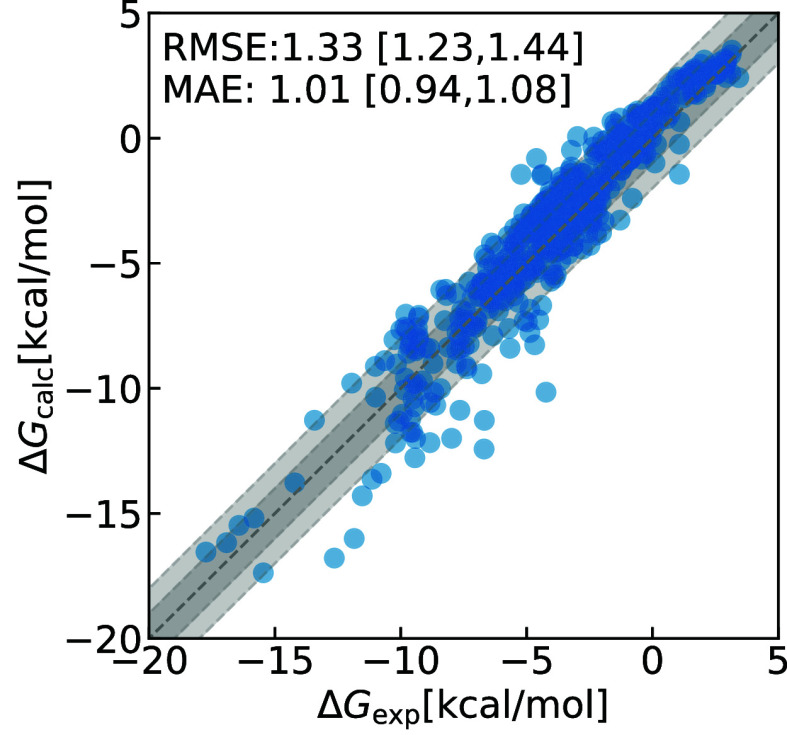
Absolute solvation free energy calculations using the OpenFF force field. Δ*G*_calc_ are the calculated values using protocol EXS, while experimental values (Δ*G*_exp_) are taken from the literature.^47^ The dark and light gray areas depict the ±1 and ±2 kcal/mol confidence interval.

The performance of the classical ASFE results for 589 molecules of the FreeSolv database calculated with the EXS protocol (OpenFF 2.0) is good. A plot comparing experimental and calculated ASFEs is shown in Figure 2. Both RMSE: 1.33 [1.23, 1.44] kcal/mol and MAE: 1.01 [0.94, 1.08] kcal/mol are low. The values given in the brackets [] indicate the 95% confidence interval obtained via bootstrapping. The RMSE and MAE obtained with OpenFF 2.0 are better than the values for 621 molecules obtained with CGenFF (RMSE: 1.76 [1.52, 2.02] kcal/mol, MAE: 1.12 [1.02, 1.23] kcal/mol)^9^ (protocol UVIE), as well as the values obtained with the AMBER general force field (GAFF)^60^ for all 642 molecules as reported in the FreeSolv database (RMSE: 1.54 [1.39, 1.70], MAE 1.11 [1.03, 1.19] kcal/mol).^47,48^ Note that some improved results for GAFF have been reported recently.^61,62^

In Figure S1 in the Supporting Information, we compare the ASFEs calculated with these three force fields by presenting kernel density estimates (KDE)^63,64^ of the deviation between experimental and calculated ASFE, δΔ*G*. These plots highlight a weakness of all three force fields which is not apparent from the RMSE and MAE: for all three, the peak of the KDE of δΔ*G* is near −1 kcal/mol, indicating that on average ASFEs predicted by all three force fields are too positive (i.e., too hydrophobic) by about 1 kcal/mol. This finding is in line with earlier observations; see, e.g., Mobley et al.^5^

### Endstate Corrections

#### Unidirectional Correction of the OpenFF ASFEs to ANI-2x/OpenFF (589 Compounds, Protocol EXS)

**Figure 3:**
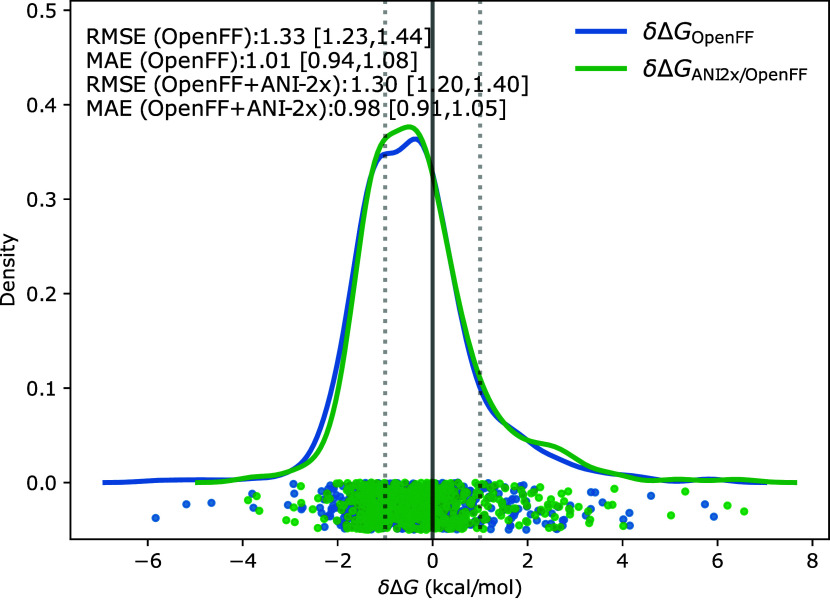
The KDE of δΔ*G* for the NNP corrected free energy estimate for the 589 compounds of the FreeSolv database. Overlay of the KDE for δ Δ*G* (eq 5) of the MM (blue line, Δ*G*_OpenFF_) and the NNP/MM (green line, Δ*G*_OpenFF/ANI2x_) free energy estimates. Additionally, the plot shows dots indicating the individual deviations from the experimental values for the MM ASFE results (blue dots) and the NNP/MM corrected ASFE results using the unidirectional correction according to protocol EXS (green dots). The gray line displays the ideal behavior, δΔ*G* = 0 with the two dotted gray lines indicating a deviation of ±1 kcal/mol.

Using the EXS protocol, we computed unidirectional Δ*G*_MM→ NNP/MM_^corr^ corrections for the 589 (out of 642) molecules in the FreeSolv database that can be described by the ANI-2x NNP (cf. Methods). As indicated in the inset of Figure 3, the RMSE and MAE values for the MM and NNP/MM results are practically identical. Similarly, the correlation with the experiment remains unchanged (Pearson correlation is 0.95 in both cases, while the Spearman correlation increased marginally from 0.94 [0.93, 0.95] to 0.95 [0.94, 0.96]). All statistical descriptors (RMSE, MAE, etc.) discussed here and later in this article are summarized in a supplementary file (jp4c01417_si_002.csv). An alternative summary of the results is shown in Figure 3. Here, we superpose the KDE of δΔ*G* (eq 5) between the experimental and calculated MM and NNP/MM corrected free energy estimates for the 589 molecules under investigation. The two KDEs are practically indistinguishable; if anything, the NNP/MM results (green curve) are shifted slightly toward more positive values.

If one studies the corrections more closely (for full details, see the data in file jp4c01417_si_002.csv in the SI), one notices that the absolute value of the correction |Δ*G*_MM→NNP/MM_^corr^| for more than half of the molecules (294) is smaller than 0.5 kcal/mol. First, this indicates that the free energy difference between the MM and NNP/MM descriptions of interactions is very similar for these solutes. Second, based on the statistical uncertainty of the corrections (see jp4c01417_si_002.csv in the SI), 0.5 kcal/mol is a rough threshold indicating whether the MM → NNP/MM correction is statistically significant. While there are certainly systems for which the statistical error is very low and, thus, corrections of, e.g., ± 0.2 or ±0.3 kcal/mol are significant, such small (absolute values of the) corrections have a marginal impact on the agreement with the experiment at best. Even if one therefore considers only solutes for which the magnitude of the correction was larger than 0.5 kcal/mol, the resulting ASFEs had improved agreement with the experimental values in only approximately 60% of the cases. Overall, both the statistical descriptors and the KDE plots show that the effect of the MM → NNP/MM correction when applied to the full data set is statistically not significant.

#### Comparison of Corrections Using Different Force Fields (Protocol UVIE and Protocol EXS, 156 Molecule Subset)

**Figure 4:**
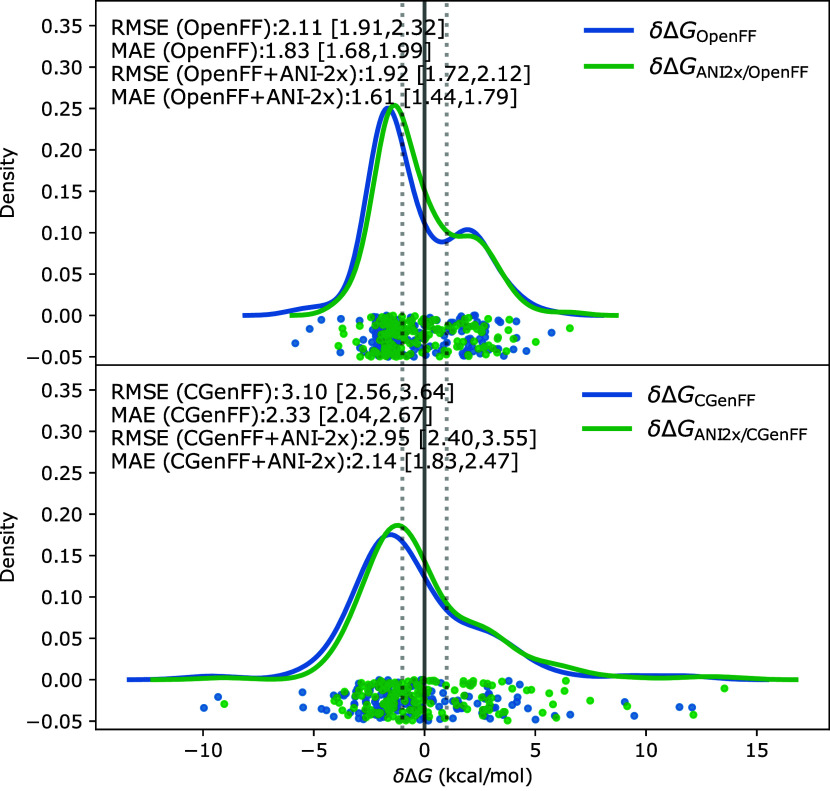
The KDE of δ Δ*G* (eq 5) for the 156 compound subset. Top: KDE of the errors obtained with OpenFF 2.0 (blue line) and for the NNP corrected results (green line). Bottom: KDE of the errors for CGenFF (blue) and NNP corrected results (green). Results for the individual molecules are shown as dots: blue for the MM, and green for the NNP corrected values.

The results of the MM → NNP/MM corrections for the subset of 156 molecules for which the computed solvation energies are in poor agreement with the experiment when using either the CGenFF or the OpenFF 2.0 force field or both are summarized in Figure 4. Using the UVIE protocol, the MAE was reduced by 0.15 kcal/mol, from 3.10 [2.56, 3.64] kcal/mol to 2.95 [2.40, 3.55] kcal/mol. While the Pearson correlation coefficient improved slightly from 0.77 [0.69, 0.83] to 0.80 [0.72, 0.87], there was no change in the Spearman correlation (0.76 [0.66, 0.83] before and 0.76 [0.64, 0.83] after the correction). Applying the NNP correction to the ASFEs obtained with OpenFF (protocol EXS) gave a similar trend. The RMSE and MAE were reduced from 2.11 [1.91, 2.32] and 1.83 [1.68, 1.99] kcal/mol to 1.92 [1.72, 2.12] and 1.61 [1.44, 1.79] kcal/mol, respectively. The Pearson as well as the Spearman correlation improved slightly from 0.90 [0.87, 0.92] to 0.93 [0.90, 0.95] and from 0.91 [0.87, 0.93] to 0.92 [0.89, 0.94] respectively. While the numbers move slightly in the right direction, none of the improvements are statistically significant. The KDEs in Figure 4 provide similar information: for both protocols, the NNP/MM corrected results are shifted slightly toward the right, including the respective peak of the KDE, but one also sees that some results become even more too positive; i.e., one sees more green than blue dots toward positive values.

Table 1 provides some information in how many cases the NNP correction (Δ*G*_MM →NNP/MM_^corr^) improved the agreement with the experimental values. In both protocols, this was the case for slightly over 60% of the solutes. However, even for this reduced subset of molecules, the (absolute value of the) correction is <0.5 kcal/mol in most cases; |Δ*G*_MM →NNP/MM_^corr^| is larger than 0.5 kcal/mol for only about 25% of the compounds (37 for CGenFF, 46 for OpenFF). For these molecules, applying the MM → NNP/MM correction improves the agreement with experiment in almost 70% of the cases, a slightly higher percentage compared to the entire 156 molecule subset.

**Table 1:** Comparison of Improvements for the 156 Compound Subset

	CGenFF	OpenFF
Corrections improving the results [%]	62	63
Percentage of molecules with |Δ*G*^corr^| > 0.5 kcal/mol [%]	24	29
Percentage thereof improving results [%]	68	67

#### Comparing Unidirectional with Bidirectional and MFES Results

In protocol UVIE, we computed the MM → NNP/MM correction not only unidirectionally, i.e., by using Jarzynski’s equation, but also carried out equilibrium simulations at the NNP/MM level of theory and backward switches in the NNP/MM → MM direction. Hence, we also calculated Δ*G*_MM↔NNP/MM_^corr, Crooks^ using Crooks’ equation. Therefore, we could investigate deviations of the unidirectional from bidirectional results. Out of the 156 compounds studied using the UVIE protocol the deviation between the Jarzynski and Crooks results, Δ_*Crooks*_ = |Δ*G*_MM↔NNP/MM_^corr, Crooks^ – Δ*G*_MM→NNP/MM_^corr^|, was larger than 1 kT only for 10 compounds, either in the gas phase or in aqueous solution, or both. For the three solutes, Δ_*Crooks*_ was larger than 2 kcal/mol. Full details, together with the 2D structures of the ten molecules, are shown in Figure S3.

When using protocol UVIE, overlap between forward and backward work distributions was monitored routinely. We observed that there was no overlap between the distributions of forward and backward work values in the three cases where Δ_*Crooks*_ > 2 kcal/mol plus one additional case. Poor or no overlap between forward and backward work distributions raises doubts about the reliability of even the Crooks results. Therefore, for these ten molecules we also computed the correction free energy by MFES (Δ*G*_MM↔NNP/MM_^corr, MFES^) and inspected Δ_MFES_ = Δ*G*_MFES_^corr^ – Δ*G*_MM↔NNP/MM_^corr, Crooks^, the deviation between the MM → NNP/MM correction calculated with Crooks’ equation and MFES. The detailed results are also plotted in Figure S3. For the six molecules for which there is overlap, Δ_MFES_ < 1 kcal/mol and most deviations are even within the 1 kT threshold. For the four molecules without overlap, the deviation is larger, as is to be expected (bottom panel of Figure S3).

These findings indicate that unidirectional methods are not sufficient in all cases, and in selected cases, even the bidirectional Crooks result may not be fully reliable. However, we observed problems only for 10 out of 156 molecules, i.e., for well below 10% of the system studied. Replacing the unidirectional corrections with the Crooks or MFES results would lead to only negligible changes in the overall RMSE, MAE, and correlation coefficients. Furthermore, the more accurate corrections (Crooks and/or MFES) do not necessarily improve the agreement with the experiment; see the spreadsheet jp4c01417_si_003.csv in the SI.

##### Investigating Large Corrections and Poor Convergence

Since Δ*G*_MM→NNP/MM_^corr^ was negligible in many, if not most, cases, it is of some interest to investigate when the correction is likely to be sizable. Furthermore, although it affects only a few systems (cf. the previous subsection), it is important to understand when and why unidirectional approaches (Jarzynski’s equation) may fail to converge. In trying to address these questions, one should keep in mind that the NNP/MM description used in this study applies only to the solute. While the solute’s interactions are treated by ANI-2x, the solute–solvent interactions are always classical. Any analysis, therefore, has to focus on the solute, i.e., the part of the system handled by the NNP.

Studies using the indirect cycle approach to compute free energy differences at the QM/MM level of theory have shown that convergence is difficult to achieve if there are different conformational preferences at the two levels of theory.^32,34,65^ We use the shorthand “different conformational preferences” to refer to situations, where the conformations sampled preferentially or exclusively at one level of theory are rare or never sampled at the respective other level of theory. Such cases are more likely for flexible solutes. The preferred conformation(s) of a solute, however, influences its solvation free energy, so for flexible solutes Δ*G*_MM→NNP/MM_^corr^ may also be larger.

**Figure 5:**
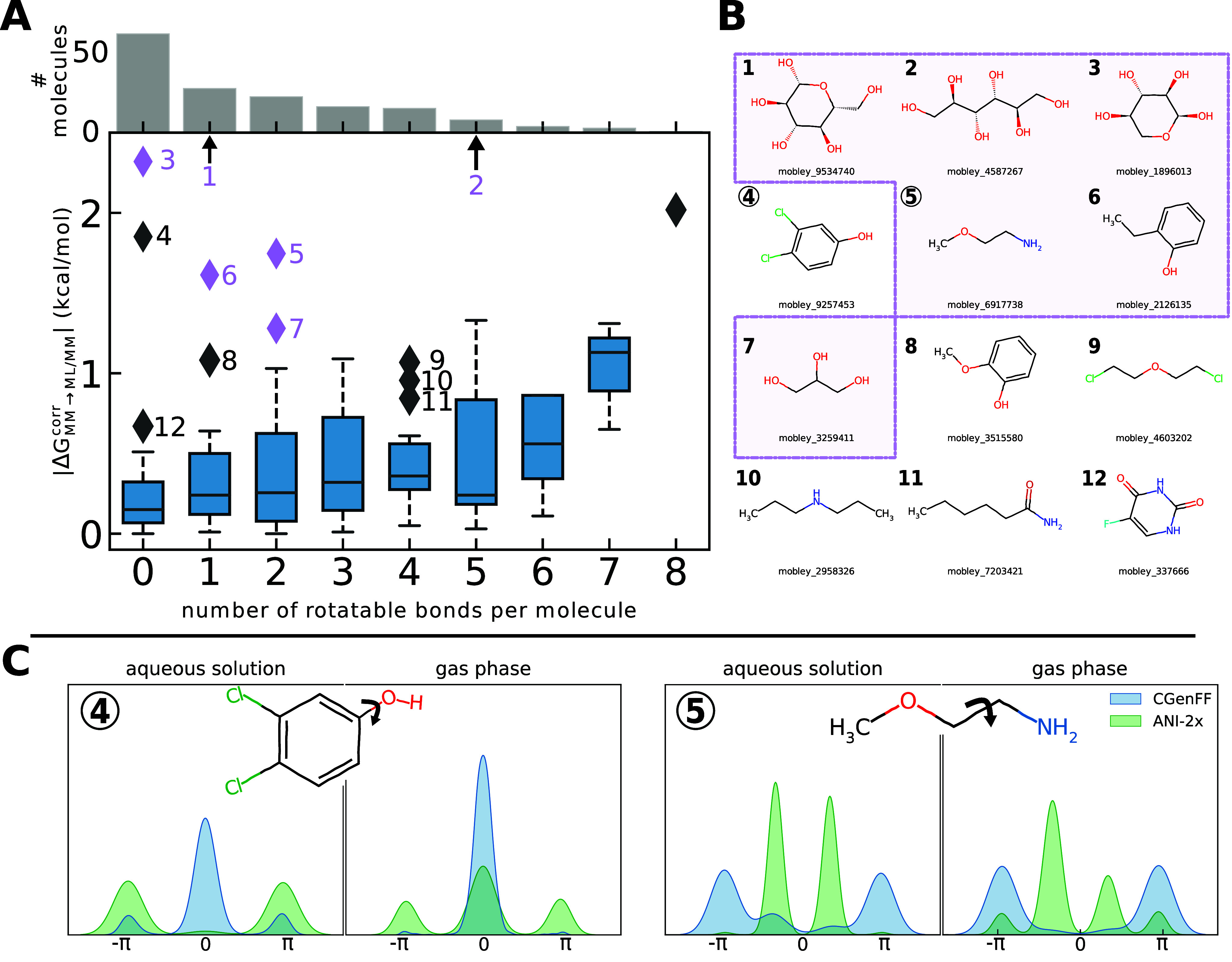
Panel A: Box plot of the unidirectional NNP correction (Δ*G*_MM→NNP/MM_^corr^) as a function of the number of rotatable bonds for the 156 compound subset using protocol UVIE (an analogous plot for EXS is shown in Figure S2 in the Supporting Information). Molecules that are outliers in terms of Δ*G*_MM→NNP/MM_^corr^ are indicated as diamonds and labeled by numbers, starting with 1 for the compound having the highest correction value. Compounds that are also outliers when using protocol EXS are colored in purple. The bars at the top of the plot indicate how many molecules have this number of rotatable bonds. Panel B: Molecular structures of the outliers 1–12. Molecules in the purple box are also outliers when using protocol EXS. Panel C: Density of the indicated dihedral angle of compounds 4 (left side) and 5 (right side), respectively, in the gas phase and in aqueous solution when using CGenFF (blue) and ANI-2x (green).

To explore this hypothesis, we grouped the MM → NNP/MM corrections obtained for the 156 compound subset and protocol UVIE according to the number of rotatable bonds *n*_*rot*_ (as reported by the *CalcNumRotatableBonds* function in the rdkit toolkit, https://www.rdkit.org/). This criterion is clearly far from perfect; e.g., aliphatic ring systems may have *n*_*rot*_ = 0, yet they are often highly flexible. In Figure 5A, Δ*G*_MM→NNP/MM_^corr^ as a function of *n*_*rot*_ is shown as box plots; all outliers (shown as diamonds) have numbers indicating the molecules in question (these are shown in Figure 5B). An analogous plot for EXS is shown in Figure S2 of the Supporting Information. Solutes that are outliers in both protocols are highlighted in purple. Ignoring for the moment the outliers, there appears to be a slight trend toward larger corrections as *n*_*rot*_ increases. Given that changing from an MM to an NNP potential energy function affects only the intramolecular interactions of the solute, the Δ*G*_MM→NNP/MM_^corr^ correction is expected to be small for rigid molecules (low *n*_*rot*_, aromatic rings). Conversely, however, the correction does not have to be significant for flexible molecules. First, if the MM and the NNP descriptions of the solute intramolecular interactions lead to similar conformational preferences, there is little reason to expect large corrections. Furthermore, even if the MM and NNP descriptions of the solute do result in different conformational preferences, the solute–solvent interactions, which are described classically throughout, may still be similar.

Analyzing some outliers provides additional insight. The two solutes with the largest Δ*G*_MM→NNP/MM_^corr^ value have *n*_*rot*_ = 1 (compound 1) and *n*_*rot*_ = 5 (compound 2). Both have also large Δ*G*_MM→NNP/MM_^corr^ values using protocol EXS (see Figure S2) and converge poorly; see the previous subsection and Figure S3. The pyranose ring of compound 1 is an example where the *n*_*rot*_ criterion fails; obviously, this cyclic structure is highly flexible, but this is not picked up by rdkit’s rotatable bond criterion. This is also the case for compound 3, which is reported as *n*_*rot*_ = 0. Compound 2, on the other hand, is obviously flexible and has a large number of rotatable bonds. On the other hand, the Δ*G*_MM→NNP/MM_^corr^ value of compound 4 (*n*_*rot*_ = 0) seems unexpected, as this is an aromatic ring. A possible explanation can be seen in Figure 5C, left panel, where the average population of the indicated C–C–O–H dihedral angle is plotted. Especially in aqueous solution, the distributions of this dihedral angle are quite different for the force field (blue) and for ANI-2x (green). The orientation of the hydroxyl group relative to the chlorine substituents may influence the solvation free energy of the molecule. An analogous plot is shown in the right panel of Figure 5C for compound 5. It has two rotatable bonds, one of which, as indicated, is populated quite differently when the solute is described by MM and NNP, respectively. Similarly to what was just discussed for compound 4, the different orientation of the hydroxyl group relative to the other substituent(s) when using MM and ANI-2x, respectively (data not shown), also seems to be the cause of the large Δ*G*_MM→NNP/MM_^corr^ corrections for compounds 6 and 8. Finally, compound 9, bis-2-chloroethyl ether, was discussed in some detail in ref (32), where it was found that the conformational preferences of the two relevant dihedral angles differ significantly between MM and the semiempirical QM method used.

## Conclusion

Utilizing the ANI-2x potential, we employed an automated protocol to correct solvation free energies obtained with the OpenFF and CGenFF force fields. When the MM → NNP/MM correction is applied to the full subset of molecules that can be described by ANI-2x, the minuscule overall improvement in free energy is statistically not significant (see Figure 3). Focusing on the subset of molecules for which the error of the ASFE is highest using the MM protocols, we can observe some improvement and large corrections for a few molecules (Figures 4 and 5). Even here, however, the changes are statistically not significant, and for the systems where the corrections are >0.5 kcal/mol, they improve the agreement with the experiment only in less than 70% of the cases.

The majority of the results presented here were calculated using unidirectional NEQ protocols, i.e., Jarzynsky’s equation. The ANI-2x potential is sufficiently fast so that we could also carry out bidirectional NEQ switching and sampling for a sizable subset of the FreeSolv (156 molecules, protocol UVIE). Only a small subset (10 out of 156) showed statistically relevant deviations between the free energy estimate based on the forward NEQ switching trajectory and the forward and reverse NEQ switching trajectories. Given the limitations of mechanical embedding (see below), using unidirectional methods to compute the MM → NNP/MM corrections seems adequate.

There are important lessons to be learned from these results: The currently available coupling between MM and ANI corresponds to mechanical embedding in QM/MM.^10,14^ The description of the solute–solvent interactions remains classical at both levels of theory (MM and NNP/MM). This explains why different results are obtained depending on the MM force field used and why the corrections are tiny in most cases. As shown, large corrections are obtained primarily for flexible solutes. We, therefore, surmise that improving free energy estimates significantly will require advanced treatment of the interaction of the small molecule with its surroundings, i.e., moving beyond mechanical embedding.^66−69^ The importance of describing solute–solvent interactions as accurately as possible when calculating solvation free energy differences has been noted previously.^70−72^ An attempt to go beyond mechanical embedding for a handful of solutes is described in the SI; the data suggest that treating solute–solvent interactions at the NNP level of theory has significant effects, but the results clearly are not converged.

As an alternative to more advanced embeddings, treatment of the entire system with the NNP is a possibility. The performance of, e.g., ANI-2x in OpenMM is sufficient to allow nanosecond simulations of solute–solvent systems consisting of the solute and up to a thousand solvent molecules. Obviously, the success of direct free energy simulations at the NNP level of theory depends on several prerequisites. First, the NNP used has to reproduce the condensed phase properties of aqueous solutions correctly. In addition, protocols for the annihilation or decoupling of the solute need to be developed to ensure that end point catastrophes are avoided.

## Data Availability

All plots shown in this paper were produced using the Jupyter-notebook available on GitHub (https://github.com/JohannesKarwou/notebooks/blob/main/combinedDataset.ipynb). The notebook also contains the calculations of all statistics reported in this paper (RMSE, MAE, Pearson correlation, and Spearman’s rank correlation) and the corresponding bootstrapped errors. Python package used in this work (release v0.3): https://github.com/wiederm/endstate_correction.

## Supporting Information Available

We describe in detail how ASFEs were calculated on the MM level of theory and how the endstate corrections to the ANI-2x potential were applied for the two respective protocols (UVIE and EXS). In Figure S2 we show the unidirectional NNP correction as a function of rotatable bonds for the 156 compounds subset using protocol EXS as well as the structures of the outliers observed. In Figure S3 we show several characteristics of the 10 compounds (of the 156 subset), for which the MM → NNP/MM correction differed by more than 1 kT when computed by Jarzynski’s and Crooks’ equation (PDF)
The ASFE on the MM and NNP/MM level for the 589 compounds of the FreeSolv database calculated using EXS are summarized in the spreadsheet *jp4c01417_si_002.csv* (CSV)
The ASFE values on the MM and NNP/MM level for the two force fields (OpenFF 2.0 and CGenFF) for the 156 compounds of the combined data set calculated with protocol EXS and UVIE are summarized in the spreadsheet *jp4c01417_si_003.csv*. In the case of the correction for CGenFF, unidirectional values are indicated with “Jar”, while bidirectional values are depicted with “Crooks” (CSV)
