# Supplementary material for: Insights and Challenges in Correcting Force Field Based Solvation Free Energies Using a Neural Network Potential
Source: J Phys Chem B. 2024 Jul 8;128(28):6693–703. doi: 10.1021/acs.jpcb.4c01417 (PMC11264272; doi:10.1021/acs.jpcb.4c01417)
Supplement: Supplementary file 1 — jp4c01417_si_001.pdf [file jp4c01417_si_001.pdf]

Insights and Challenges in Correcting Force Field Based Solvation Free Energies Using A Neural Network Potential

Johannes Karwounopoulos,[†,‡] Zhiyi Wu,[¶] Sara Tkaczyk,[§,‖] Shuzhe Wang,[¶] Adam Baskerville,[¶] Kavindri Ranasinghe,[¶] Thierry Langer,[§] Geoffrey P. F. Wood,[¶] Marcus Wieder,[*,¶,⊥] and Stefan Boresch[*,†]

†*Faculty of Chemistry, Institute of Computational Biological Chemistry, University Vienna, Währingerstr. 17, 1090 Vienna, Austria*

‡*Vienna Doctoral School of Chemistry (DoSChem), University of Vienna, Währingerstr. 42, 1090 Vienna, Austria*

¶*Exscientia plc, Schroedinger Building, Oxford, United Kingdom*

§*Department of Pharmaceutical Sciences, Pharmaceutical Chemistry Division, University of Vienna, Josef-Holaubek-Platz 2, 1090 Vienna, Austria*

‖*Vienna Doctoral School of Pharmaceutical, Nutritional and Sport Sciences (PhaNuSpo), University of Vienna, Josef-Holaubek-Platz 2, 1090 Vienna, Austria*

⊥*Open Molecular Software Foundation, Davis, California 95616, United States*

E-mail: marcus.wieder@gmail.com; stefan@mdy.univie.ac.at

Additional details for the `EXS` and `UVIE` protocols

Protocol `UVIE`— CGenFF as the classical force field

ASFE calculations with `transformato`

Here, a short summary of the protocol used in our previous work is given; for the full details, see Ref. 1: For each calculation, we generated the solvated system using the SMILES string provided by the FreeSolv database with the Python extension of Open Babel (Pybel).[2] Missing solute parameters were generated with a stand-alone version of `cgenff` (v2.5.1), based on version 4.6 of the CHARMM general force field (CGenFF).[3–7] The solutes were placed in cubic simulation boxes with a side length of $\geq$ 26 Å, which is sufficiently large to be commensurate with the default CHARMM cut-off radius of 12 Å for Lennard-Jones interactions. Coulomb interactions were calculated using the particle-mesh Ewald (PME) method[8] (Ewald $\kappa = 0.34\text{Å}^{-1}$, $32 \times 32 \times 32$ FFT grid); Lennard-Jones (LJ) interactions were calculated using a cutoff of 12 Å and the default OpenMM switching function between 10 Å and 12 Å. These steps were automated with the Python package `macha`,* which uses CHARMM scripts generated by CHARMM-GUI[9,10] as templates. When used to compute ASFEs, `transformato` provides an automated workflow to set up the serial atom insertion (SAI) approach.[11] Since electrostatic and Lennard-Jones intramolecular interactions are annihilated, the gas phase correction step (see left side of Figure 1 in the main manuscript) is required to restore both intramolecular interactions. Each ASFE calculation was repeated four times with different initial random velocities. The standard deviation was calculated from these four runs.

Treatment of virtual particles on chlorine atoms

Recent versions of the CGenFF force field[5,6] use virtual particles to better describe the halogen bond, a specific non-covalent interaction between a halogen atom and another electronegative atom, driven by a localized positive electrostatic region known as the ”sigma

*`https://github.com/akaupang/macha`

hole".[7] While these virtual particles can be handled straightforwardly in the `transformato` workflow, they must not be present during the NEQ switching simulations from the MM to the ANI-2x level of theory. For the 24 molecules that contain a chlorine atom, we, therefore, had to ensure the closing of the alchemical cycle by introducing a suitable intermediate state. We calculated the free energy difference between the original state (fully charged virtual particle attached to each chlorine present) and an intermediate state, where the virtual particles are present, but their charges have been transferred to the corresponding chlorine atom. In this intermediate state, the massless virtual particles do not contribute to the system's free energy. Hence, the virtual particles can be safely removed when carrying out the endstate correction from MM to ANI-2x.

Nonequilibrium switching simulations

Setup of the forward (MM→NNP/MM) switching simulations: Four independent 5 ns MD simulations were carried out, using the Langevin integrator with a target temperature of 303K and a friction coefficient of 1/ps. Pooling the trajectories of the 4 simulations of the physical endstate for each of the systems and excluding the first 30% of each run as equilibration, we collected 28,000 coordinate sets from which the forward switching protocol (MM → NNP/MM) was initialized.

Setup of the backward (NNP/MM→MM) switching simulations: At the NNP/MM endstate, a single 10 ns trajectory was generated for each system (all other simulation parameters as just described). Again, the first 30% were discarded, resulting in 7,000 coordinate frames.

Switching simulations: From the pool of initial configurations, 300 coordinate sets were selected randomly (with replacement) as the starting point of the forward and backward NEQ switching simulations, both in the gas phase and in solution.

Protocol `EXS`— OpenFF as the classical force field

ASFE with openmmtools

Molecules were parametrized using Biosimspace[12] and the OpenFF-2.0 force field.[13] The solvated systems were created by solvating the ligand in a 40Å^3 cubic water box. In solution, electrostatic interactions were computed using PME[8] with a short-range cutoff of 10 Å; no cutoff was used in the gas phase. Bond lengths involving hydrogens were constrained to their parameter value in all simulations. OpenMM 8.0[14] was used to perform all simulations. Before starting an MD simulation, both the gas phase and solvated structure were minimized. Simulations were performed using the LangevinMiddleIntegrator implemented in openmmtools[15] with a target temperature of 300 K and a friction coefficient of 1/ps, using a time step of 2 fs. Each system in the gas phase was equilibrated in NVT for 1 ns. Each solvated system was equilibrated for 1 ns in the NPT ensemble with a Monte Carlo barostat at a target pressure of 1 atm. The ASFE calculation consisted of two phases: In solution, interactions were decoupled/annihilated using 26 λ-windows. First, intra- and intermolecular electrostatic interactions were scaled to zero using an equidistant λ-spacing of 0.1 (i.e., electrostatic interactions were annihilated in 11 steps). Then, the van der Waals interactions were decoupled with a non-equidistant scheduling (using 0.05 spacing until $\lambda = 0.5$, then continuing with a 0.1 spacing). Soft core potentials were used as described in Equation 13 of Ref. 16. Because of the mixed annihilation/decoupling scheme, the gas phase corrections only had to restore intramolecular electrostatic interactions. Each λ-window was simulated for 10 ns. Errors were estimated from the single repeat equilibrium simulations via the bootstrapping functionality of MBAR as implemented in `pymbar`.[17]

Nonequilibrium switching simulations

Simulations of the physical endstates were repeated, treating the solute as fully flexible (i.e., only water molecules were kept rigid by constraints). During these simulations, 5,000 frames

were saved. From these coordinates, 300 were selected randomly (with replacement) and used to initialize 5 ps forward switching simulations (MM $\rightarrow$ NNP/MM).

An attempt to go beyond mechanical embedding

As described in the main manuscript, correcting hydration free energies to the NNP/MM level of theory led only to statistically insignificant changes compared to the MM results; i.e., the magnitude of the endstate correction was small in most cases. We strongly suspect that this is a consequence of the mechanical embedding[18] currently used for the NNP/MM coupling. Since the performance of ANI-2x is sufficiently fast, we attempted to go beyond mechanical embedding for a handful of compounds as follows. All calculations described below are based on protocol UVIE; the starting point are the trajectories saved during NNP/MM simulations (mechanical embedding) needed for the bidirectional calculation (Crooks' equation) of the end state corrections. Given a set of coordinates, the solute–solvent interaction energy can always be obtained as the difference

$$\mathrm{U_{inter}} = \mathrm{U(full)} - \mathrm{U(water)} - \mathrm{U(solute)} \tag{1}$$

Here, U(full) is the total energy, U(water) the energy of just the waters, and U(solute) the energy of the solute with the waters removed. For each frame saved during the NNP/MM simulations, the necessary energies were recomputed (1) using the NNP/MM coupling and mechanical embedding as during the production calculations, and (2) using ANI-2x for the full system. We, thus, extracted interaction energies $\mathrm{U_{inter}^{MM}}$ and $\mathrm{U_{inter}^{NNP}}$ at the MM and NNP levels of theory, respectively. We then estimated a correction based on the NNP interaction energy according to

$$\Delta G_{\mathrm{inter}} = -k_B T \ln \left\langle \exp\left(-\frac{\mathrm{U_{inter}^{NNP}} - \mathrm{U_{inter}^{MM}}}{k_B T}\right)\right\rangle_{MM} \tag{2}$$

The correction ΔG_{inter} was then added to the endstate corrected hydration free energy obtained with mechanical embedding.

The results are summarized in Table S1. While, with one exception, the magnitude of the endstate corrections resulting from mechanical embedding (column EC) was small (< 0.5 kcal/mol), that of the endstate corrections including interaction energies at the NNP level of theory is much larger. However, in most cases the resulting hydration free energy (column ASFE) is in worse agreement with experiment than the MM and the NNP/MM result obtained with mechanical embedding.

Table S1 also includes the standard deviation $\sigma(\Delta \text{U}_{\text{inter}})$ of the difference in interaction energies $\Delta \text{U}_{\text{inter}} = \text{U}_{\text{inter}}^{\text{NNP}} - \text{U}_{\text{inter}}^{\text{MM}}$. As one sees, $\sigma(\Delta \text{U}_{\text{inter}}) > 4$ kcal/mol in all cases. In Ref. 19 it was shown that results obtained from Zwanzig's or Jarzinsky's equation become unreliable if the standard deviation of the energy differences / work values exceeds $4\ k_B T \approx 2.4$ kcal/mol at room temperature. This is the case for all compounds considered; hence, ΔG_{inter} is likely subject to large systematic errors.

Table S1: Results obtained by correcting the solute–solvent interaction energy from MM to NNP. All free energies are in kcal/mol.

ID[a]	name[b]	EXP[c]	MM[d]	EC[e]	NNP/MM[f]	ΔG_{inter}[g]	$\sigma(\Delta \text{U}_{\text{inter}})$[h]	ASFE[i]
2410897	ethanamine	-4.5	-1.34	-0.52	-1.84	0.56	4.3	-1.28
3968043	cyclopentanone	-4.7	-2.05	0.18	-1.87	2.41	4.78	0.54
6239320	cyanuric acid	-18.06	-30.14	-0.07	-30.21	1.14	5.92	-29.07
6266306	azetidine	-5.56	-3.1	-0.34	-3.44	1.63	4.06	-1.81
6917738	2-methoxyethanamine	-6.55	-3.38	-1.35	-4.73	-2.58	4.3	-7.31
7261305	hydrazine	-9.3	-6.83	-0.02	-6.85	-0.91	5.1	-7.76
8048190	acetamide	-9.71	-7.86	-0.16	-8.02	-9.49	4.11	-17.51

[a] ID used in FreeSolv;[20,21] [b] common name; [c] experimental hydration free energy reported in FreeSolv; [d] MM hydration free energy obtained using protocol UVIE; [e] end state correction as obtained using protocol UVIE (mechanical embedding only); [f] hydration free energy after reweighting to ANI-2x/MM, mechanical embedding, protocol UVIE ; [g] correction ΔG_{inter} resulting from replacing the MM interaction energy by the NNP interaction energy (see Eq. 2); [h] standard deviation of $\Delta \text{U}_{\text{inter}} = \text{U}_{\text{inter}}^{\text{NNP}} - \text{U}_{\text{inter}}^{\text{MM}}$; [i] hydration free energy after applying the correction for the interaction energy as described.

Nevertheless, some conclusions can be drawn from the data summarized in Table S1. First, endstate corrections obtained with solute–solvent interactions at the NNP level of

theory are indeed larger than those obtained from mechanical embedding only. Unfortunately, achieving convergence seems non-trivial; the values found for $\sigma(\Delta \mathrm{U}_{\mathrm{inter}})$ are too high, making the use of Zwanzig's equation unreliable. It is, in principle, possible to calculate the correction $\Delta G_{\mathrm{inter}}$ using non-equilibrium work methods, but this will require nontrivial adaptions and performance enhancements of our NNP/MM coupling codebase.

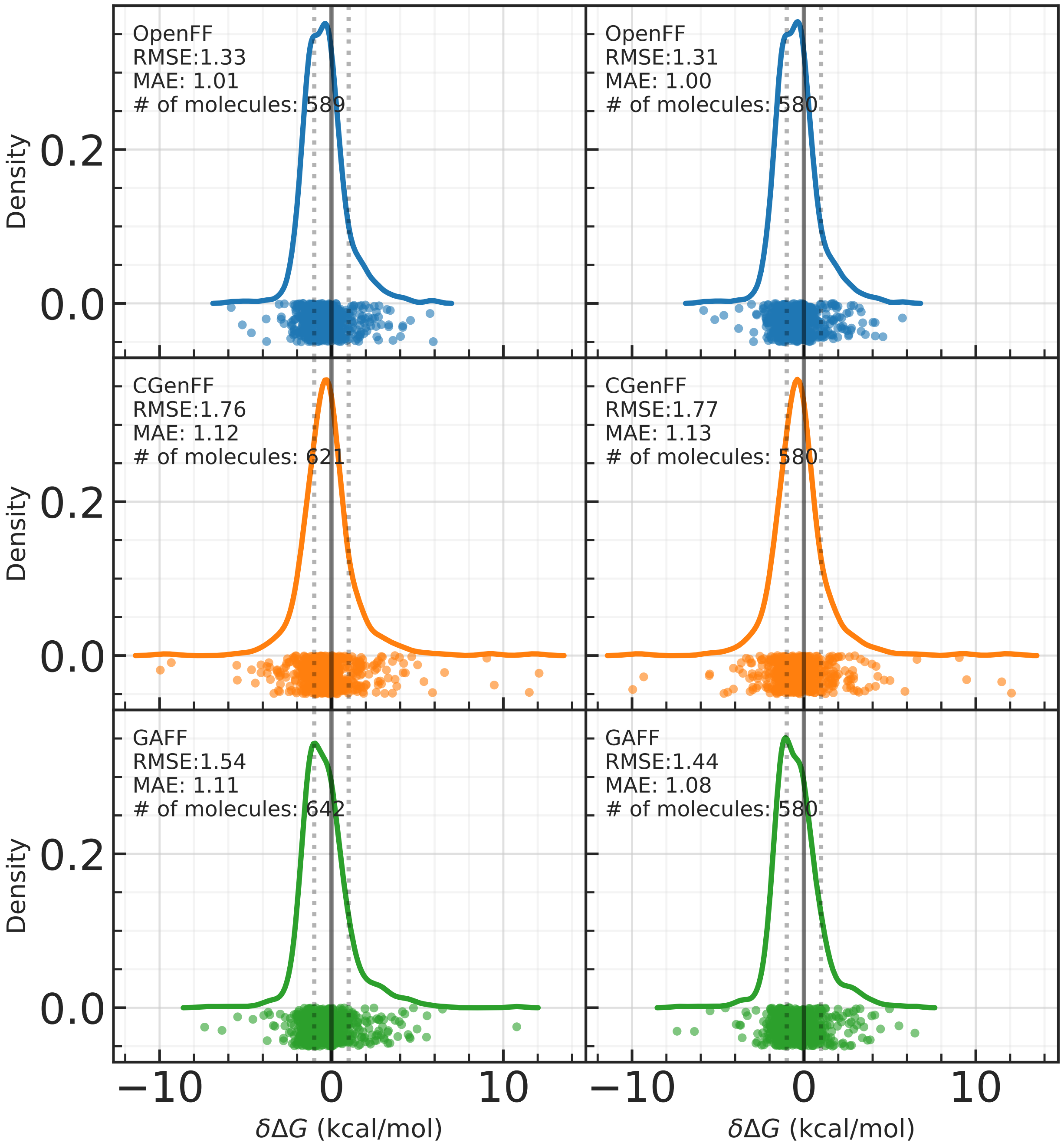

Figure S1: **Kernel Density Estimation (KDE) of $\delta\Delta G = \Delta G_{exp} - \Delta G_{calc}$, the error in calculated ASFEs compared with the experimental data, using three classical force fields.** Top: OpenFF (this work); middle: CGenFF;[1] bottom: GAFF.[20,21] The plots on the left are for all available data for the respective force field (OpenFF: 589, CGenFF: 621, GAFF: 642). Plots on the right display the results for the 580 molecules for which calculated ASFE values are available across all three force fields.

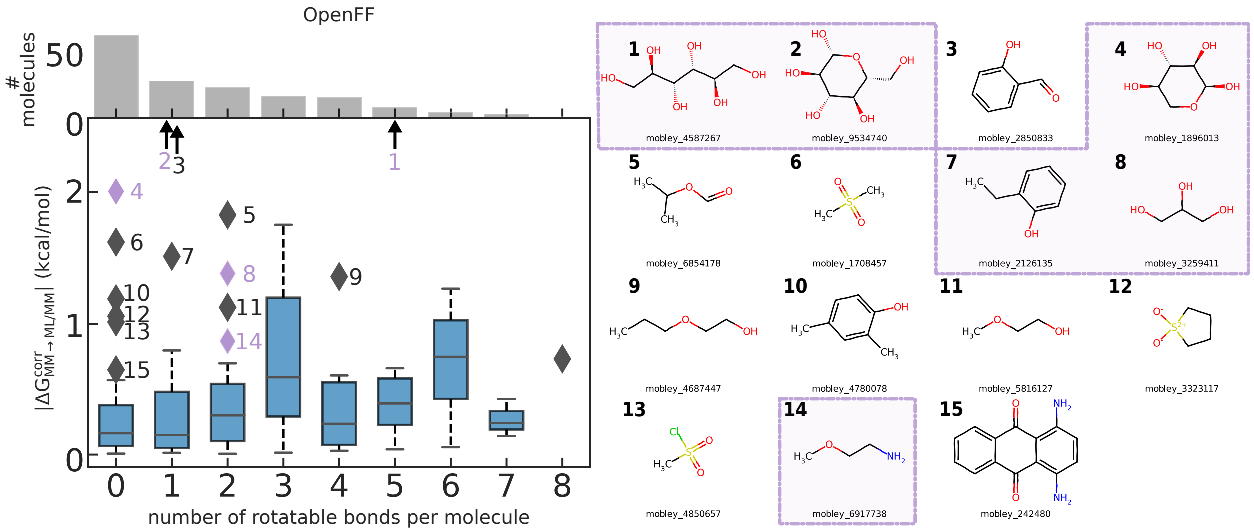

Figure S2: **Unidirectional NNP correction ($\Delta G^{corr}_{MM \rightarrow NNP/MM}$) as a function of the number of rotatable bonds for the 156 compound subset using protocol EXS.** The molecules are numbered, starting with one (1) for the compound having the highest correction value. Outliers observed for both force fields (CGenFF and OpenFF) are highlighted in purple. See Figure 5 in the main manuscript for the analogous plot using the CGenFF force field.

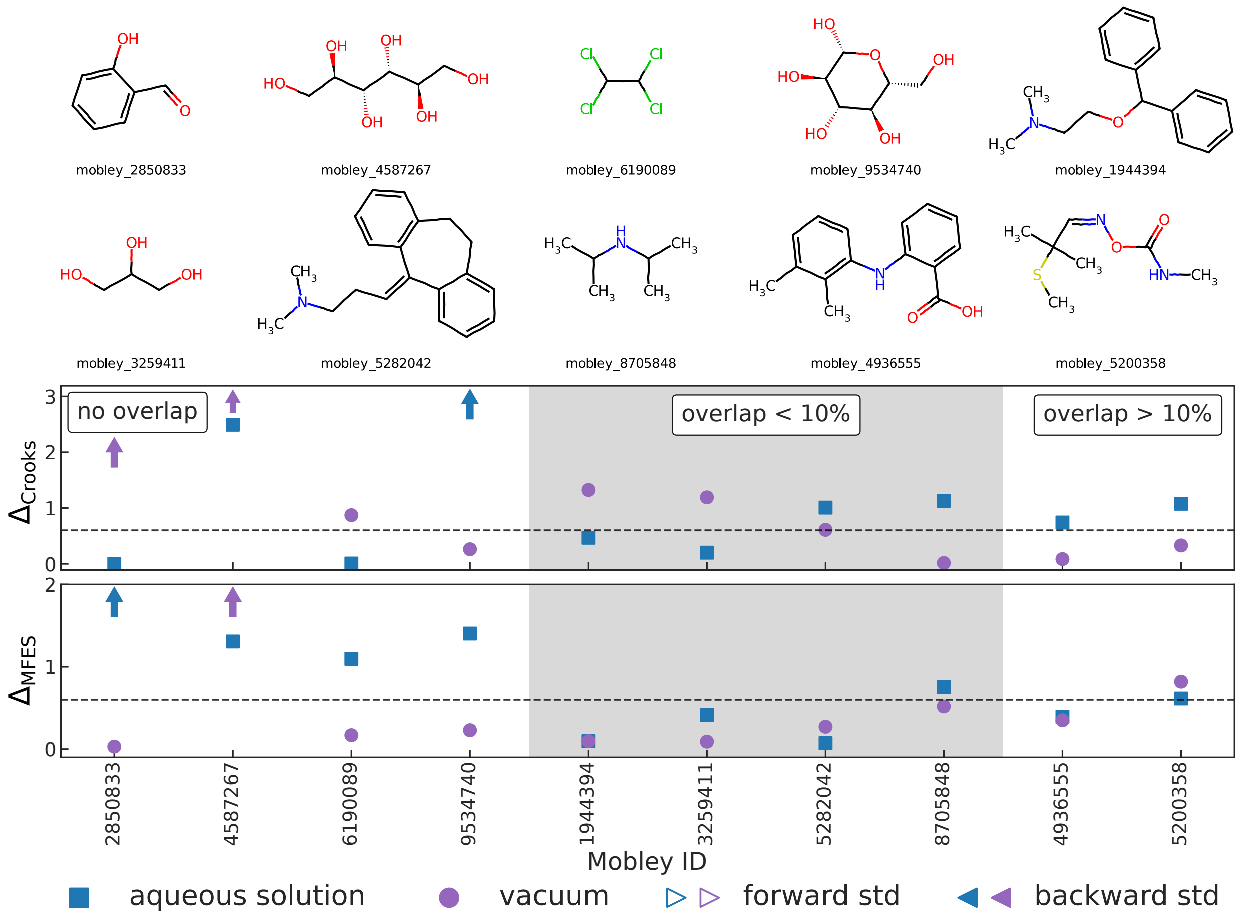

Figure S3: Several characteristics of the 10 compounds out of the 156 molecule subset for which the MM→NNP/MM correction differed by more than 1 kT when computed by Jarzynski's and Crooks' equation, either in vacuum (purple symbols) or in the aqueous environment (blue symbols), or both. The entries are sorted according to the overlap of their forward and backward work distributions: those with no overlap in either environment are presented on the left, compounds with less than 10% overlap in at least one environment are displayed in the middle (shaded in gray), and compounds with more than 10% overlap are shown on the right. All free energy and work-related quantities are in kcal/mol.
Top panel: 2D structures of the ten compounds. Middle panel: Absolute value of the deviation between the unidirectional Jarzynski and bidirectional Crooks results, $\Delta_{\text{Crooks}} = \left|\Delta G^{\text{corr,Crooks}}_{\text{MM}\leftrightarrow\text{NNP/MM}} - \Delta G^{\text{corr}}_{\text{MM}\rightarrow\text{NNP/MM}}\right|$. Bottom panel: Absolute deviation between values calculated from NEQ simulations using the two-sided Crook's equation and values calculated by MFES ($\Delta_{\text{MFES}} = \Delta \text{G}^{\text{corr}}_{\text{MFES}} - \Delta G^{\text{corr,Crooks}}_{\text{MM}\leftrightarrow\text{NNP/MM}}$). Arrows in the middle and bottom panels indicate off-scale values, i.e., >3 kcal/mol for Δ_{Crooks} (middle panel) or >2 kcal/mol for Δ_{MFES} (bottom panel).

References

(1) Karwounopoulos, J.; Kaupang, Å.; Wieder, M.; Boresch, S. Calculations of Absolute Solvation Free Energies with Transformato – Application to the FreeSolv Database Using the CGenFF Force Field. *J. Chem. Theory Comput.* **2023**, *19*, 5988–5998.

(2) O'Boyle, N. M.; Morley, C.; Hutchison, G. R. Pybel: A Python wrapper for the OpenBabel cheminformatics toolkit. *Chem. Cent. J.* **2008**, *2*, 1–7.

(3) Vanommeslaeghe, K.; Hatcher, E.; Acharya, C.; Kundu, S.; Zhong, S.; Shim, J.; Darian, E.; Guvench, O.; Lopes, P.; Vorobyov, I.; Mackerell, A. D. CHARMM general force field: A force field for drug-like molecules compatible with the CHARMM all-atom additive biological force fields. *J. Comput. Chem.* **2010**, *31*, 671–690.

(4) Yu, W.; He, X.; Vanommeslaeghe, K.; MacKerell, A. D. Extension of the CHARMM general force field to sulfonyl-containing compounds and its utility in biomolecular simulations. *J. Comput. Chem.* **2012**, *33*, 2451–2468.

(5) Vanommeslaeghe, K.; Raman, E. P.; MacKerell, A. D. Automation of the CHARMM General Force Field (CGenFF) II: Assignment of Bonded Parameters and Partial Atomic Charges. *J. Chem. Inf. Model.* **2012**, *52*, 3155–3168.

(6) Vanommeslaeghe, K.; MacKerell, A. D. Automation of the CHARMM general force field (CGenFF) I: Bond perception and atom typing. *J. Chem. Inf. Model.* **2012**, *52*, 3144–3154.

(7) Gutiérrez, I. S.; Lin, F.-Y.; Vanommeslaeghe, K.; Lemkul, J. A.; Armacost, K. A.; Brooks, C. L.; MacKerell, A. D. Parametrization of halogen bonds in the CHARMM general force field: Improved treatment of ligand–protein interactions. *Bioorg. Med. Chem.* **2016**, *24*, 4812–4825.

(8) Essmann, U.; Perera, L.; Berkowitz, M. L.; Darden, T.; Lee, H.; Pedersen, L. G. A smooth particle mesh Ewald method. *J. Chem. Phys.* **1995**, *103*, 8577.

(9) Lee, J. et al. CHARMM-GUI Input Generator for NAMD, GROMACS, AMBER, OpenMM, and CHARMM/OpenMM Simulations Using the CHARMM36 Additive Force Field. *J. Chem. Theory Comput.* **2016**, *12*, 405–413.

(10) Jo, S.; Kim, T.; Iyer, V. G.; Im, W. CHARMM-GUI: A web-based graphical user interface for CHARMM. *J. Comput. Chem.* **2008**, *29*, 1859–1865.

(11) Boresch, S.; Bruckner, S. Avoiding the van der Waals endpoint problem using serial atomic insertion. *J. Comput. Chem.* **2011**, *32*, 2449–2458.

(12) Hedges, L. O.; Mey, A. S.; Laughton, C. A.; Gervasio, F. L.; Mulholland, A. J.; Woods, C. J.; Michel, J. BioSimSpace: An interoperable Python framework for biomolecular simulation. *Journal of Open Source Software* **2019**, *4*, 1831.

(13) Boothroyd, S. et al. Development and Benchmarking of Open Force Field 2.0.0: The Sage Small Molecule Force Field. *J. Chem. Theory Comput.* **2023**, *19*, 3251–3275.

(14) Eastman, P. et al. 2023, DOI: 10.48550/ARXIV.2310.03121.

(15) John Chodera et al. choderalab/openmmtools: 0.23.1 (accessed 2024-05-21). 2023; https://zenodo.org/records/8102771.

(16) Pham, T. T.; Shirts, M. R. Identifying low variance pathways for free energy calculations of molecular transformations in solution phase. *J. Chem. Phys.* **2011**, *135*, 034114.

(17) Shirts, M. R.; Chodera, J. D. Statistically optimal analysis of samples from multiple equilibrium states. *J. Chem. Phys.* **2008**, *129*, 124105.

(18) Galvelis, R.; Varela-Rial, A.; Doerr, S.; Fino, R.; Eastman, P.; Markland, T. E.; Chodera, J. D.; De Fabritiis, G. NNP/MM: Accelerating Molecular Dynamics Simulations with Machine Learning Potentials and Molecular Mechanics. *J. Chem. Inf. Model* **2023**, *63*, 5701–5708.

(19) Boresch, S.; Woodcock, H. L. Convergence of single-step free energy perturbation. *Mol. Phys.* **2017**, *115*, 1200–1213.

(20) Mobley, D. L.; Guthrie, J. P. FreeSolv: a database of experimental and calculated hydration free energies, with input files. *J. Comput. Aided Mol. Des.* **2014**, *28*, 711–720.

(21) Duarte Ramos Matos, G.; Kyu, D. Y.; Loeffler, H. H.; Chodera, J. D.; Shirts, M. R.; Mobley, D. L. Approaches for Calculating Solvation Free Energies and Enthalpies Demonstrated with an Update of the FreeSolv Database. *J. Chem. Eng. Data* **2017**, *62*, 1559–1569.
